# Imaging in patients with acute dyspnea when cardiac or pulmonary origin is suspected

**DOI:** 10.1259/bjro.20220026

**Published:** 2023-02-02

**Authors:** Ruxandra-Iulia Milos, Carmen Bartha, Sebastian Röhrich, Benedikt H. Heidinger, Florian Prayer, Lucian Beer, Christian Wassipaul, Daria Kifjak, Martin L Watzenboeck, Svitlana Pochepnia, Helmut Prosch

**Affiliations:** 1 Department of Biomedical Imaging and Image-guided Therapy, Medical University of Vienna, Vienna, Austria; 2 Service des Urgences, CHC MontLegia, Liége, Belgium; 3 Department of Radiology, UMass Memorial Medical Center and University of Massachusetts Chan Medical School, Worcester, United States

## Abstract

A wide spectrum of conditions, from life-threatening to non-urgent, can manifest with acute dyspnea, thus presenting major challenges for the treating physician when establishing the diagnosis and severity of the underlying disease. Imaging plays a decisive role in the assessment of acute dyspnea of cardiac and/or pulmonary origin. This article presents an overview of the current imaging modalities used to narrow the differential diagnosis in the assessment of acute dyspnea of cardiac or pulmonary origin. The current indications, findings, accuracy, and limits of each imaging modality are reported.

Chest radiography is usually the primary imaging modality applied. There is a low radiation dose associated with this method, and it can assess the presence of fluid in the lung or pleura, consolidations, hyperinflation, pneumothorax, as well as heart enlargement. However, its low sensitivity limits the ability of the chest radiograph to accurately identify the causes of acute dyspnea.

CT provides more detailed imaging of the cardiorespiratory system, and therefore, better sensitivity and specificity results, but it is accompanied by higher radiation exposure. Ultrasonography has the advantage of using no radiation, and is fast and feasible as a bedside test and appropriate for the assessment of unstable patients. However, patient-specific factors, such as body habitus, may limit its image quality and interpretability.

Advances in knowledge

This review provides guidance to the appropriate choice of imaging modalities in the diagnosis of patients with dyspnea of cardiac or pulmonary origin.

## Introduction

Acute dyspnea, or shortness of breath, is a major public health issue, and is one of the most common complaints of patients who present to the emergency department (ED), accounting for almost four million ED visits annually in the United States.^1^ Patients with dyspnea make up to 5% of ED presentations, around 10% of ward admissions, and 20% of intensive care unit (ICU) admissions.^2^ According to the American Thoracic Society, dyspnea is defined as “a subjective experience of breathing discomfort that consists of qualitatively distinct sensations that vary in intensity”.^3^ Dyspnea is considered acute when it develops over hours to days, but some patients may present with an acute exacerbation of a chronic condition, which can be triggered by a new concomitant illness or a worsening of the original disease.

Dyspnea is an umbrella term, and one of the main difficulties confronting the clinician is to diagnose and determine the severity of the underlying disease, as dyspnea can include life-threatening conditions, such as tension pneumothorax, or non-urgent disorders, such as deconditioning. Epidemiologic studies of dyspneic patients have shown that the most common ED diagnoses included pneumonia (~25%), heart failure (~18%), chronic obstructive pulmonary disease (COPD) exacerbation (~15%), and asthma (~10%), with an overall in-hospital mortality of 5%.^4^ With advanced age, there is an increased incidence of dyspnea of a cardiac origin, COPD exacerbation, and pulmonary embolism, as well as an increase in patients with more than two diagnoses or exacerbations of chronic disease,^5,6^ as well as an increase in in-hospital mortality.^2,5^ Dyspnea covers a broad differential diagnosis that requires rapid evaluation with special attention to several aspects of the history, physical examination, blood biomarkers, and radiological evaluation. The common causes of acute dyspnea can be divided into those of a pulmonary origin (*e.g.* pneumothorax, pulmonary embolism, airflow limitation, aspiration, pneumonia), a cardiac origin (*e.g.* myocardial ischemia or infarction, heart failure, arrhythmias, valvular heart disease, cardiac tamponade), a metabolic origin (*e.g.* ketoacidosis, poisoning, anemia), and others, including sepsis and a psychogenic origin. The challenge is to establish a timely and cost-effective diagnosis. Radiological examination plays a decisive role in the assessment of acute dyspnea of a cardiac and/or a pulmonary origin and is less important for other causes of dyspnea.

In the present paper, we discuss the radiological assessment of dyspnea of a cardiac and/or a pulmonary origin and describe how the underlying pathologies can be diagnosed using the most common imaging modalities.

## Overview of imaging modalities

## Chest radiograph

## Suspected cardiac origin

**Figure 1.**
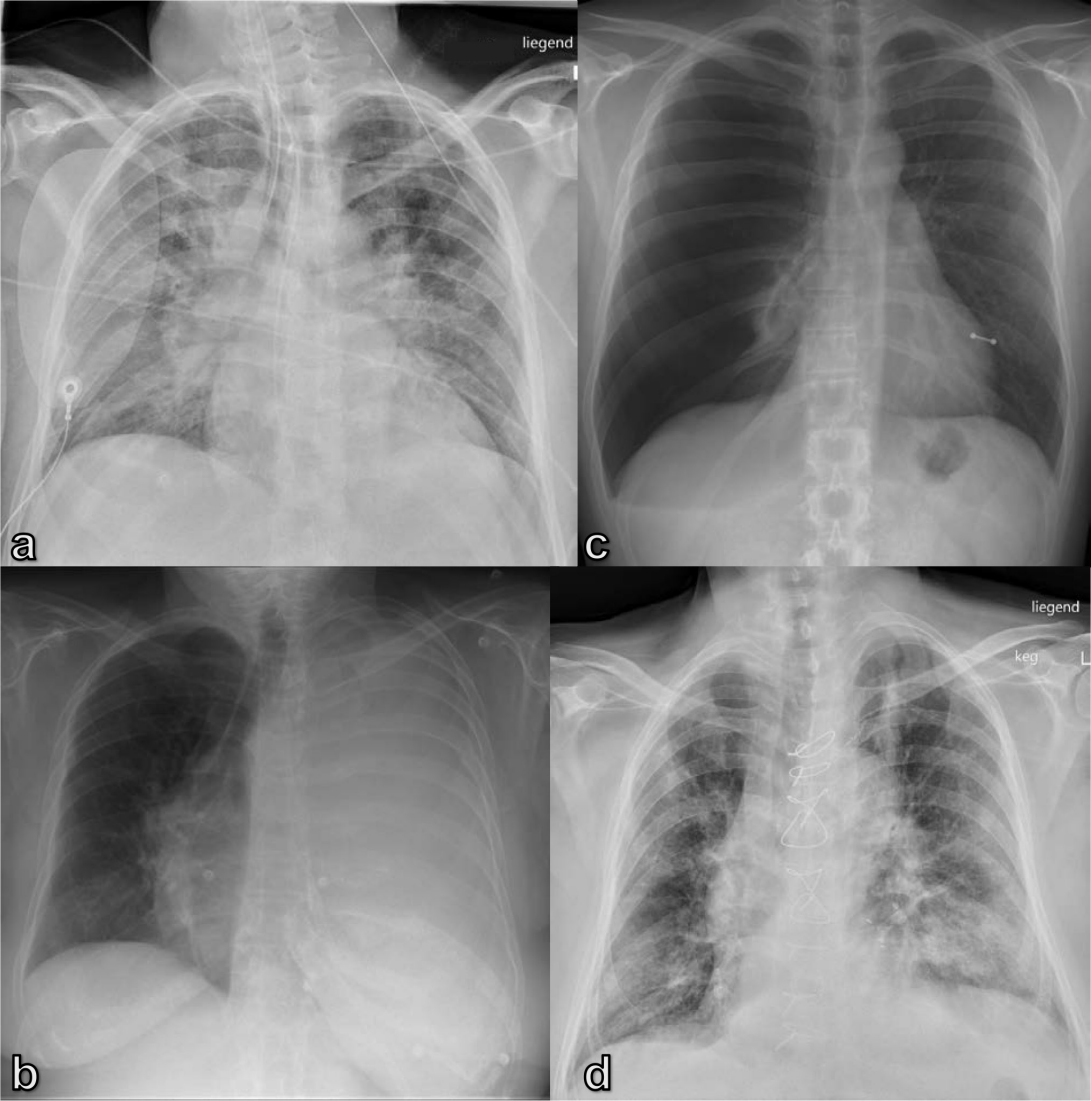
Diagnostic CXRs in dyspneic patients: (a) 55-year-old male patient with posterior wall infarct, followed by ventricular fibrillation and resuscitation with signs of pulmonary edema of cardiac origin; (b) 76-year-old female patient with known metastasized breast cancer and malignant pleural effusion on the left side; (c) 34-year-old male patient with a spontaneous tension pneumothorax on the right side; (d) 78-year-old male patient with COVID-19 pneumonia showing scattered air space opacities and areas of interstitial thickening in both lungs. No pleural effusion. CXR, chest X-ray.

For patients with dyspnea of a suspected cardiac origin, the American College of Radiology (ACR) recommends that diagnostic imaging should start with chest radiographs (CXRs), followed by transthoracic echocardiography (TTE).^7^ Similarly, the recently published European Guidelines recommend that the diagnostic workup of suspected new onset acute heart failure (AHF) should include ECG and echocardiography, if possible. In addition, CXR and lung ultrasound may be used to confirm an AHF diagnosis, especially when natriuretic peptide testing is not available.^8^ Chest radiography alone has a low sensitivity, but a high specificity in diagnosing congestive heart failure, with better results according to the level of the physician’s training, with an accuracy of up to 95%.^9,10^ Pulmonary venous congestion with upper lung zone flow redistribution, interstitial or alveolar edema manifesting as peribronchial cuffing, septal lines (the so-called Kerley B lines), airspace opacification with a batwing distribution, cardiomegaly, and bilateral pleural effusions are useful indicators of a primarily cardiac-related dyspnea on CXR (Table 1 and Figure 1A). Furthermore, semi-quantitative analysis of CXR using a congestion score index provided good diagnostic value with which to diagnose AHF in dyspneic patients.^11^ Nonetheless, a normal CXR does not rule out cardiac disease.^9,10^

**Table 1.** Radiographic findings on chest radiographs in conditions that can accompany acute dyspnea

Condition	Radiographic finding	Chest radiograph value for diagnosis
Pulmonary edema of cardiac origin	Upper lung zone flow redistribution Lung interstitial or alveolar edema ± peribronchial cuffing ± septal (Kerley) lines ± airspace opacification/batwing distribution Bilateral pleural effusion Cardiac enlargement	Diagnostic, taken together
Pneumothorax	Visible visceral pleural edge Absence of peripheral lung markings ± subcutaneous emphysema	Diagnostic
Tension pneumothorax	Findings of pneumothorax **and** Contralateral mediastinal displacement Ipsilateral increased intercostal spaces Depression of the hemidiaphragm ± lung collapse	Diagnostic
Pneumonia	Airspace opacification patchy/confluent Air bronchograms Complications: pleural collection, cavitation	Diagnostic in context
Pulmonary embolism	Enlarged pulmonary artery (Fleischner sign) Peripheral wedge opacity (Hampton hump) Regional oligemia (Westermark sign) Abrupt tapering/cutoff of a pulmonary artery (knuckle sign) ± pleural effusion	Suggestive in context, rare findings
Asthma	No diagnostic features Hyperinflation Bronchial wall thickening: peribronchial cuffing	Useful to exclude complications
COPD	No diagnostic features, possible findings: Hyperinflation Decreased peripheral bronchovascular markings Increased lung lucency ± bulla ± prominence of the hilar vessels in pulmonary hypertension	Useful to exclude complications
Pleural effusion	Unilateral/bilateral dense opacification forming a meniscus at the costophrenic angle ± mediastinal displacement if large volume	Diagnostic
Pericardial effusion/tamponade	Diagnostic features for more than 200 ml fluid: Globular heart enlargement (water bottle sign) Radiolucent epicardial and pericardial fat stripes in between radiopaque fluid stripe on lateral CXR (Oreo cookie sign) Widening of subcarinal angle	Suggestive in context

COPD, chronic obstructive pulmonary disease; CXR, chest radiograph.

CXR can indicate pericardial effusion (Table 1), calcifications, or air; however, the imaging modalities of choice for the evaluation of pericardial disease are echocardiography followed by cardiac CT or MRI, if indicated.^7^

## Suspected pulmonary origin

For patients who present with acute dyspnea due to diseases of the respiratory system, CXR plays a crucial role in the diagnostic process, and, in many cases, radiographs are the only imaging modality required to establish the diagnosis.

**Figure 2.**
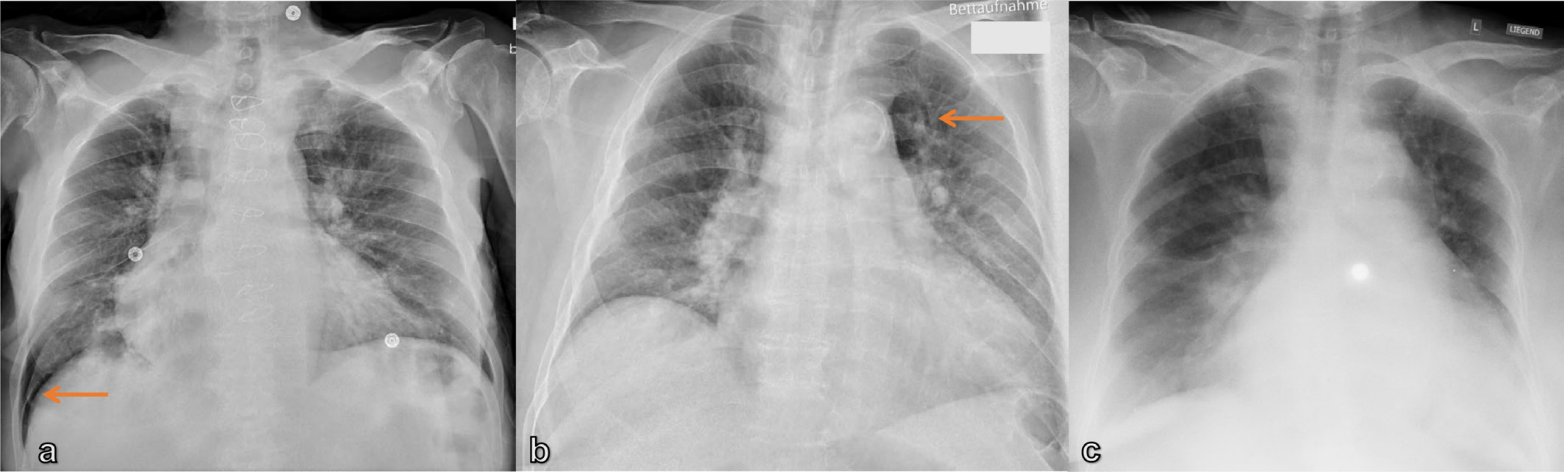
(a) Supine CXR of an 80-year-old male dyspneic patient with pneumothorax and deep sulcus sign (arrow); (b) 72-year-old male dyspneic patient with acute asthma exacerbation; supine CXR showing a small area of increased radiolucency in the para-aortic region (arrow) and the appearance of sharp edges of the descending aorta; (c) 49-year-old female patient 11 years after kidney transplant with dyspnea; bedside CXR shows signs of pleural effusion with thickening of the horizontal fissure, veil-like increased density of the hemithorax on the right side, and an enlarged heart silhouette.

A pneumothorax or pleural effusion, sufficient to cause acute dyspnea, are usually visible on CXR and show characteristic radiological findings (Table 1). In the case of pneumothorax, when air accumulates between the two pleural layers, the visceral pleura become visible as a thin dense line with no bronchovascular structures beyond it (Figure 1C). Subcutaneous emphysema and pneumomediastinum may also be present. When a CXR is acquired in a supine position, the pleura line may not always be present. When the air in the pleural cavity accumulates anterolaterally outlining the deep costophrenic angle the deep sulcus sign can be visualized (Figure 2A). However, there are some other indirect signs suggestive of the diagnosis, such as increased radiolucency of the paracardial region and the appearance of sharp edges of the mediastinum, heart, and subcutaneous tissues, or the visibility of the anteroinferior edge of the lung (Figure 2B).^12^ Diagnosis of tension pneumothorax is based on clinical signs manifesting with mediastinal shift (tracheal deviation, displaced apex), increasing respiratory distress, cyanosis, hypotension and tachycardia. On CXR, the tension pneumothorax is established in the presence of a contralateral shift of the heart and mediastinum, flattening of the cardiac profile, lowering and eversion of the hemi-diaphragm, ipsilateral increased intercostal spaces with protrusion of the parietal pleura, reduced size of the superior vena cava, and lung collapse.

Pleural effusion can be detected as a dependent opacity with a meniscus-shaped contour at the costophrenic angle and obliteration of the diaphragmatic contour. In the case of massive effusion, the entire hemithorax can be filled and the mediastinum can be shifted contralaterally (Figure 1B). On the bedside CXR, it is extremely easy to underestimate the real amount of the effusion, due to the redistribution of fluid in the more declivous parts of the thorax. Some radiological signs, however, enable a diagnosis, such as thickening of fissures, the blurring of the diaphragmatic profile, the opacification of the costophrenic angles, and the veil-like increased density of the hemithorax, with the vascular tree still visible (Figure 2C).^12^ In supine patient, the apical posterior zones are more declivous and can accumulate large amount of pleural effusion, thus producing on CXR an opacification at the apex. Atelectasis may mimic effusions, accounting for most false-positive findings.^13^ Still, pleural effusions that are large enough to cause dyspnea are detected in 92% of cases on bedside CXR and they can be excluded with high confidence.^13^

Chest radiographs play a key role in the initial diagnosis and management of immunocompetent patients who present with dyspnea due to respiratory infection.^14^ The primary role of imaging in these patients is to aid in the diagnosis or exclusion of bacterial pneumonia, and thus, to identify patients who would benefit from antibiotic therapy.^14^ The Infectious Disease Society of America/American Thoracic Society (IDSA/ATS) guidelines from 2007 state that the diagnosis of community-associated pneumonia (CAP) requires, in addition to suggestive clinical features, the demonstration of an infiltrate on CXR or other imaging modalities.^15^ The alveolar space becomes filled with inflammatory cells and pus, replacing aerated lung and results in a pulmonary opacity (formerly referred to as “infiltrate”). Initially patchy, it may become confluent as infection progresses. The air-filled bronchi cause the so-called “air bronchograms,” defined as “air-filled (low-attenuation) bronchi on a background of opaque (high-attenuation) airless lung”.^16^ CXR in patients with pneumonia may be unremarkable in about one-third of patients despite suggestive symptoms.^17^ In addition, chronic pathologies, such as organizing pneumonia or chronic eosinophilic pneumonia presenting as an opacity, may be misinterpreted as pneumonia. Using CT as the gold-standard, a multicenter study demonstrated a poor sensitivity of 43.5% and a positive-predictive value of 27% for CXR for the detection of pulmonary opacities in patients who present to the ED with acute cardiopulmonary symptoms.^18^ Conversely, in a study on elderly patients with a clinical suspicion of CAP, CXR missed the diagnosis in only 9.4% of these patients, when compared with CT.^19^ Dehydration is an important factor in hampering the signs of pneumonia on the chest radiograph.^20^ Despite the discrepancy about the sensitivity of chest radiographs for CAP, which ranges from 46 to 77% compared to CT as the gold-standard,^20^ CXR is still recommended as the routine primary imaging modality in immunocompetent patients who are likely to have pneumonia, as it can establish the diagnosis and differentiate it from other conditions with similar symptoms, such as self-limited viral infection.^14^ It should not be used alone, but in conjunction with the clinical context.

During the coronavirus disease 2019 (COVID-19) pandemic, rapid diagnosis was critically important for treatment decisions and for instituting the appropriate isolation measures, especially when healthcare facilities were confronted with a high caseload. Imaging was particularly useful in symptomatic patients with a high clinical suspicion for COVID-19 and negative COVID-19 test results, and also to rapidly triage patients before definitive COVID-19 test results were available.^21–24^ Furthermore, chest radiographs were recommended for patients with COVID-19 and moderate to severe disease, regardless of COVID-19 test results, in an environment where access to CT was limited.^22^ The most commonly observed COVID-19 findings on CXR are air space opacities, including consolidation and ground-glass opacities, reticular abnormalities with bilateral distribution in the mid-lung field or basally, with a peripheral predominance(Figure 1D).^25,26^

**Figure 3.**
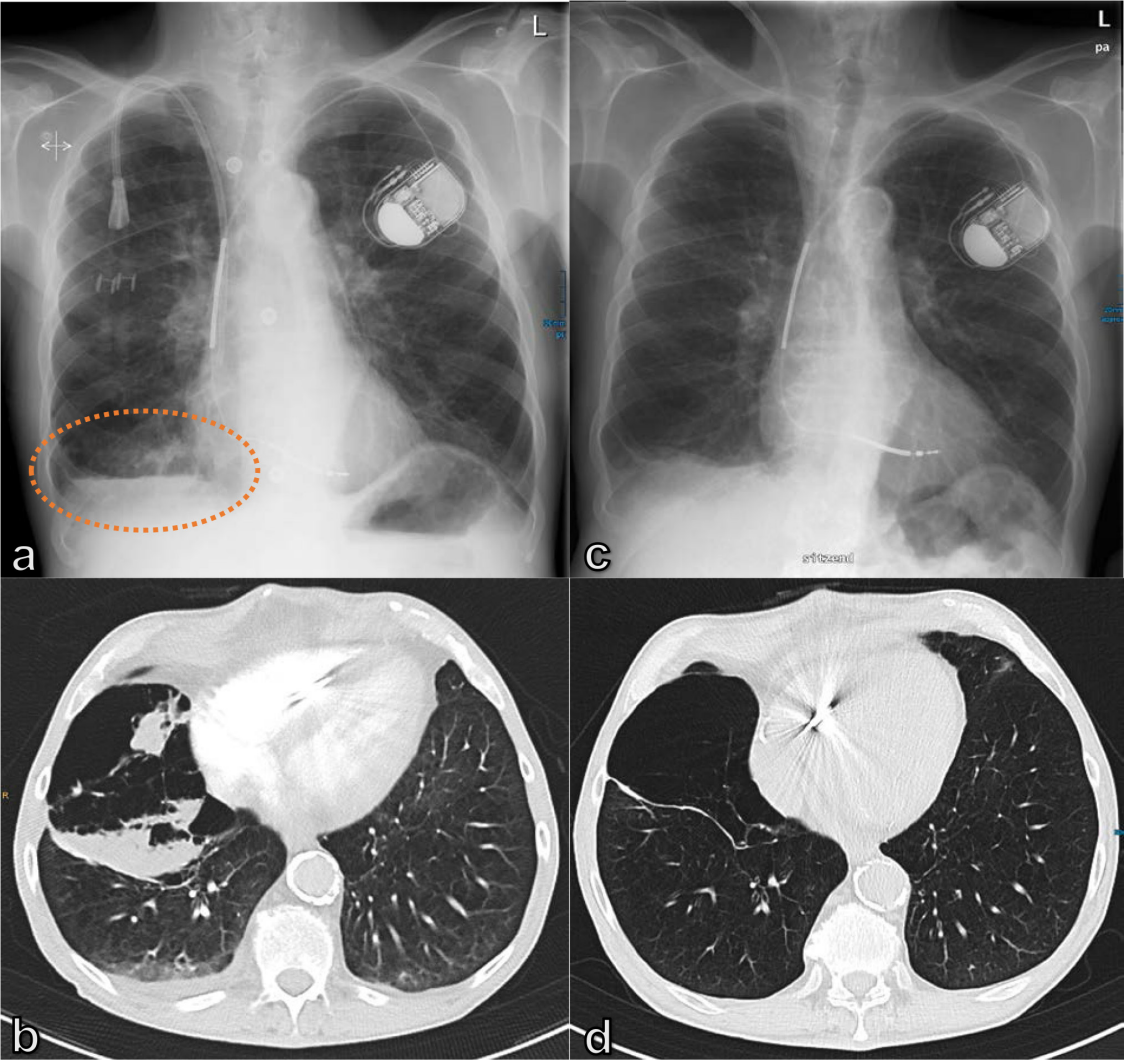
65-year-old male patient with COPD IV with massive aggravated dyspnea and tachypnea over the 3 days prior to admission: (a) CXR presenting a newly developed non-specific small opacity and gas-fluid level above the right diaphragm (circle), compared to an older image (c), while chest CT (b) depicts a newly demarcated consolidation in the dependent areas of the bullous parenchymal destruction of the middle lobe, as an inflammatory superinfection. (d) CT examination taken previously. COPD, chronic obstructive pulmonary disease; CXR, chest X-ray.

Chest radiographs are appropriate for the initial imaging of patients with a complicated acute asthma exacerbation, or acute COPD exacerbation, mainly to exclude complications, such as pneumothorax (Figure 2B) and pneumonia (Figure 3),^14^ as many patients with mild or moderate COPD and most patients with asthma have an unremarkable chest radiograph.

## Chest CT

A multicentric prospective study that investigated the role of CT in modifying physician decision-making in patients who present to the ED with chest pain and/or dyspnea showed that CT results changed the leading diagnosis in 42% of cases and CT helped confirm or exclude at least 95% of alternative diagnoses.^27^ Also, the diagnostic confidence increased and admission decision changed in 20% of cases.^27^ Dyspnea, together with chest pain, was the most common indication for CT of the chest in the ED in 75% of cases, with the most frequent pathology being pulmonary embolism at 10%, followed by pleural effusion, congestive heart failure, and pneumonia each at 6%.^28^

## Suspected cardiac origin

Coronary CT angiography (CCTA) demonstrated an excellent ability to rule out acute coronary syndrome with a high degree of confidence in low- and intermediate-risk patients.^7,29,30^ It has also been demonstrated that CCTA has a high diagnostic accuracy to exclude clinically significant coronary artery disease in patients with non-ST-segment elevation acute coronary syndrome.^31^ Patients with a normal CCTA do not require additional diagnostic testing.^32^ CCTA is less useful in patients with known coronary artery disease in the setting of severe calcifications (high calcium score) or in presence of stents. The limitations of CCTA include artifacts from an elevated or irregular heart rate that may overestimate stenosis.^7,29^

**Figure 4.**
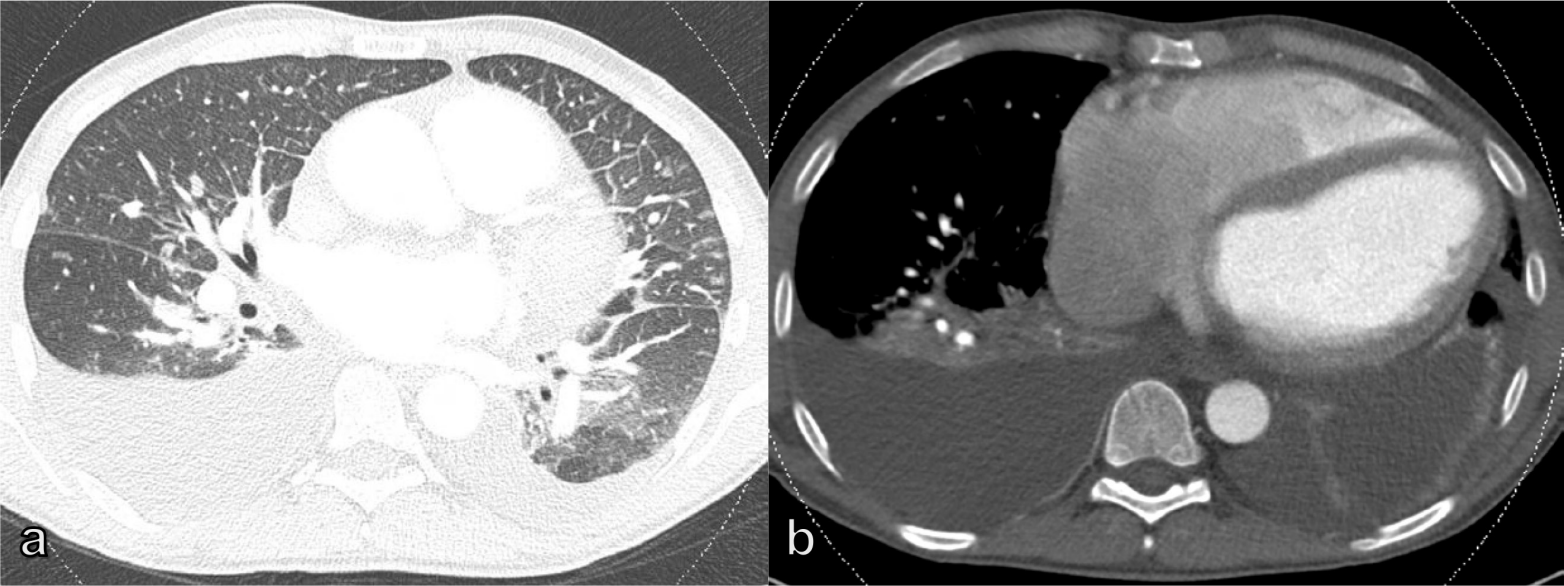
43-year-old male patient with renal insufficiency, hypertension, and signs of AHF: enlarged heart, bilateral interlobular thickening, bilateral pleural effusion, and bilateral ground-glass opacification. AHF, acute heart failure.

Chest CT is not a recommended test for AHF.^8^ However, patients with acute dyspnea often undergo CT as the primary examination in the search for a pulmonary embolism or a suspected pulmonary infection. A prospective study on patients with acute dyspnea showed that five CT signs seemed sufficient to assess the risk of AHF in the acute setting (Figure 4): enlarged heart; bilateral interlobular septal thickening; bilateral pleural effusion; increased vascular diameter; and bilateral ground-glass opacifications, with two or more signs making the AHF diagnosis almost certain.^33^ Another recent publication demonstrated that the ratio and the difference between the attenuation of the right ventricle and left ventricle have a high sensitivity for the diagnosis of AHF in ED patients who are undergoing CT pulmonary angiography and do not demonstrate a pulmonary embolism.^34^ Cardiac tamponade can be a life-threatening condition that necessitates urgent therapy. The diagnostic is usually confirmed with echocardiography. However, to identify the cause of the tamponade, and most important, to rule out a potential aortic dissection with hemopericardium, a CT angiography of the aorta is required.^35^

## Suspected pulmonary origin

**Figure 5.**
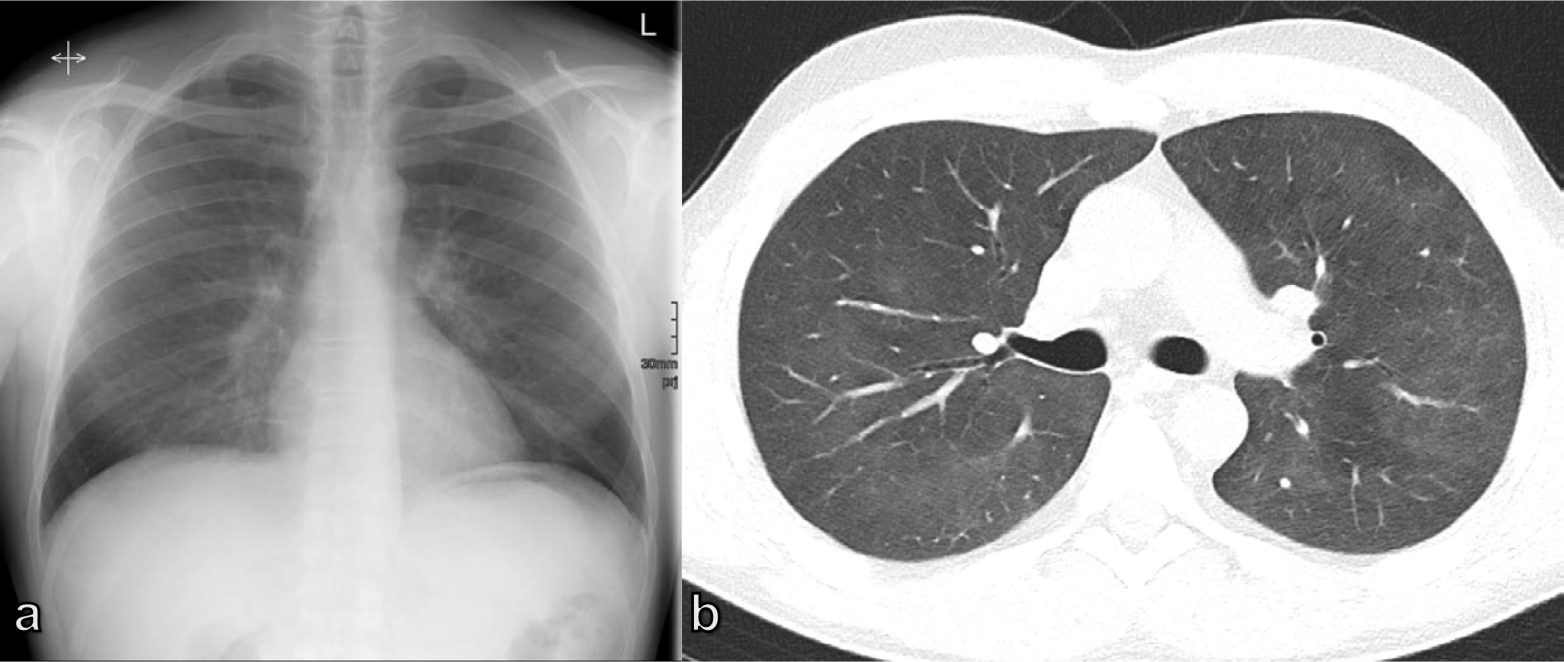
40-year-old male dyspneic patient after kidney transplant and with B-cell lymphoma under chemotherapy and an unremarkable CXR. On CT, diffuse ground-glass opacities as a radiological manifestation of Pneumocystis jirovecii pneumonia. CXR, chest X-ray.

**Figure 6.**
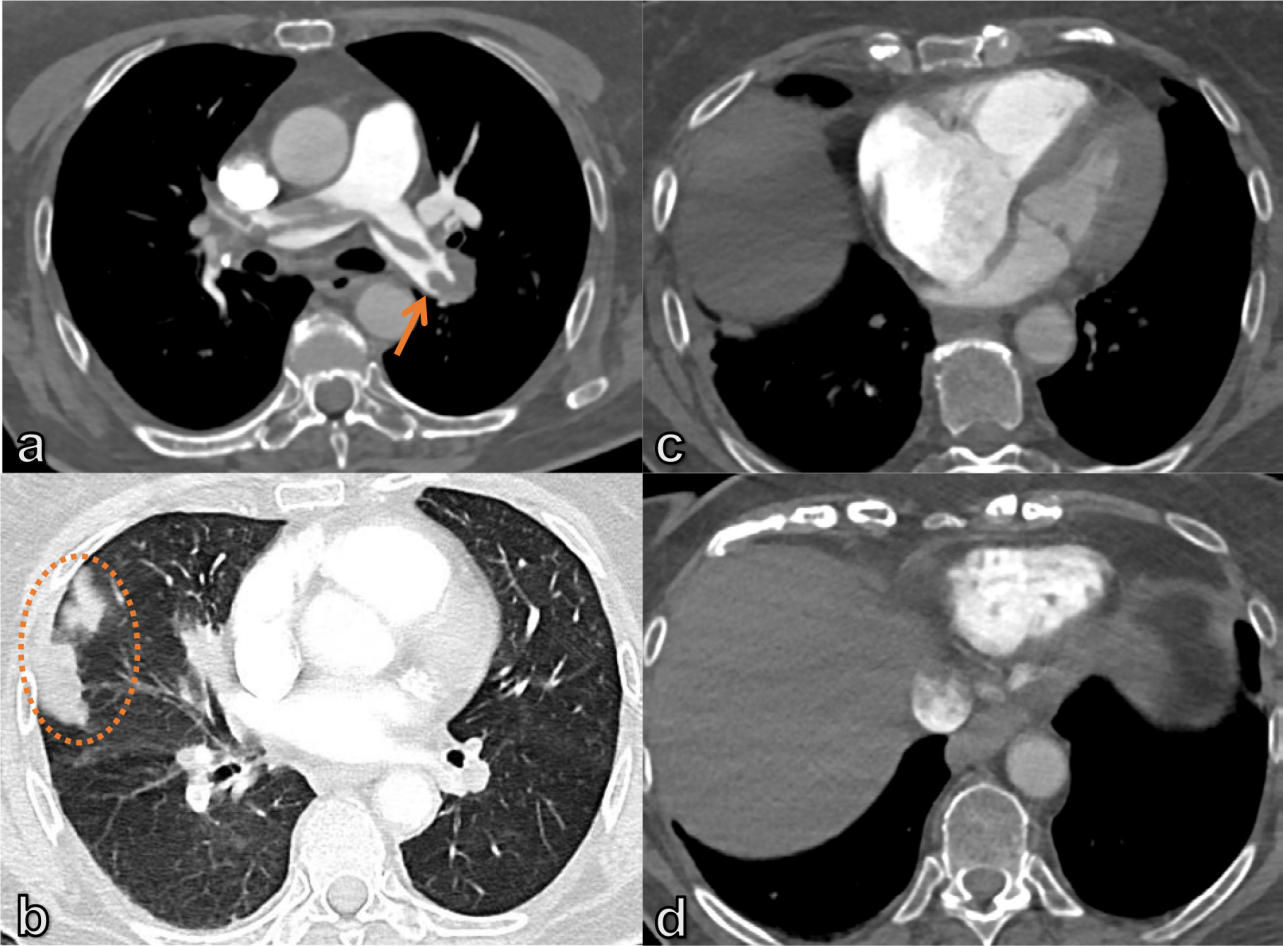
79-year-old female patient with dyspnea due to bilateral and centrally located acute pulmonary embolism with signs of right ventricle dysfunction and contrast reflux to the inferior vena cava (note the acute angle of the embolus with the vessel, arrow), as well as peripheral infarcted lung parenchyma (circle).

CT is more sensitive and specific than CXR for the detection of subtle pulmonary findings. The use of CT in patients with dyspnea of a suspected pulmonary origin is indicated in several scenarios, such as immunocompromised individuals with a clinical suspicion of pneumonia but equivocal or normal CXR (Figure 5),^36^ better characterization of abnormal but non-specific chest radiography findings (Figure 3), or the suspicion of pulmonary embolism (Figure 6).

Pulmonary infections in immunocompromised individuals comprise nearly 75% of all pulmonary complications and can progress rapidly and take a potentially fatal course.^36^ Although CXR is the initial imaging modality indicated early in the evaluation of immunocompromised patients with suspicion of a pulmonary infection, due to its superior sensitivity for subtle pulmonary abnormalities, chest CT is required if the radiograph is normal, inconclusive, or non-specific, but clinical suspicion for disease of a pulmonary origin is high.^36^ A prototype example is Pneumocystis jirovecii pneumonia in immunocompromised patients with potentially unremarkable CXR, but with presence of diffuse ground-glass opacities (Figure 5), reticular opacities, and pneumatoceles on chest CT. Furthermore, in certain immunocompetent patients in whom pulmonary infection can become life-threatening, such as those of an advanced age, or who have significant comorbidities such as organic brain disease, chest CT is recommended when the initial chest radiograph is negative or equivocal.^14^ Thus, CT is considered a reasonable alternative to empiric antibiotic therapy.^15^ In severe cases, chest CT is able to demonstrate the overall extent of disease and the presence of complications, such as abscess formation or parapneumonic effusion, and serve as a guide for pleural drainage.^14^

**Figure 7.**
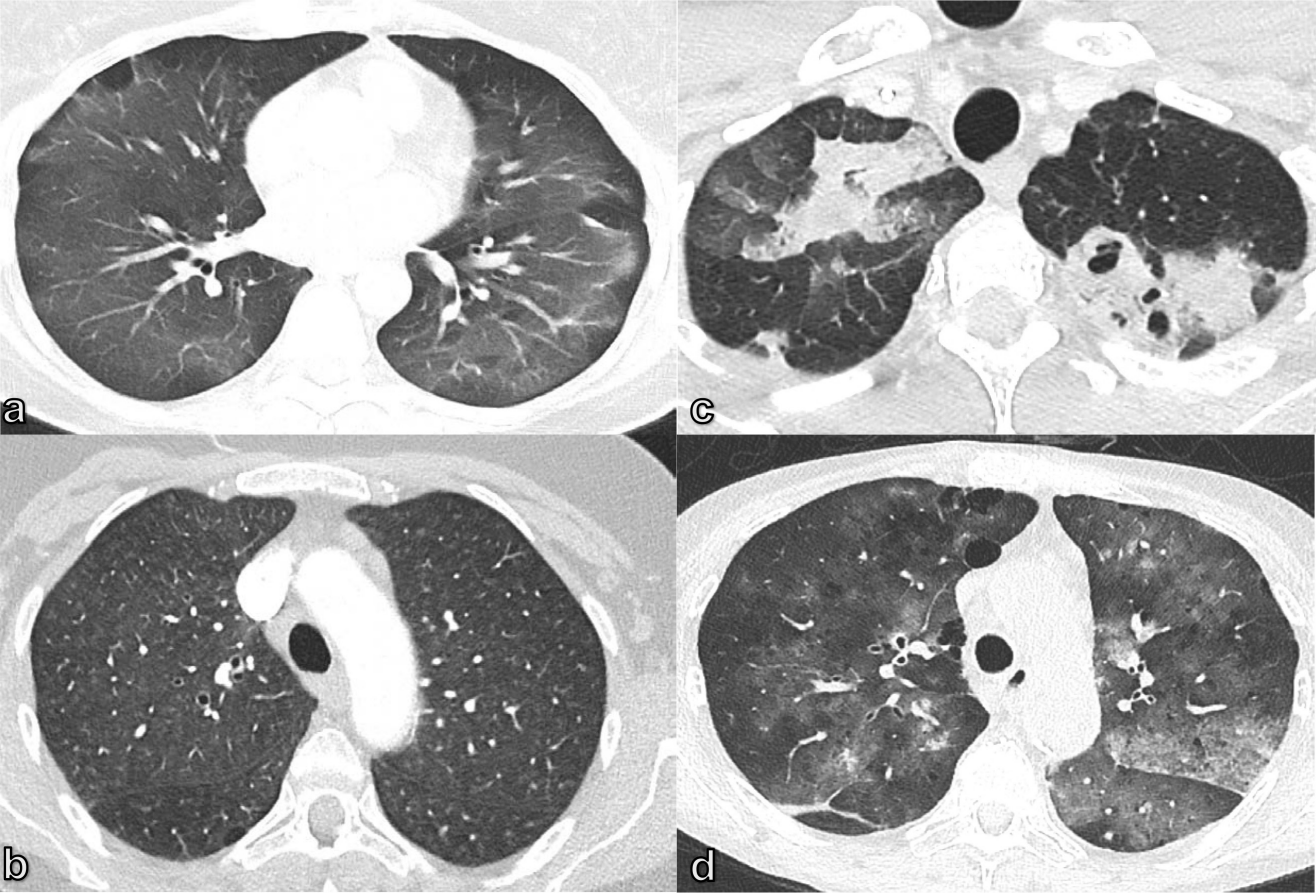
Diagnostic chest CT in dyspneic patients: (a) 48-year-old female patient with inhalation trauma and extensive ground-glass opacities as signs of permeability (non-cardiogenic) edema; (b) 52-year-old female patient with ill-defined ground-glass centrilobular nodules indicative of hypersensitivity pneumonitis; (c) 36-year-old male patient with endocarditis after tricuspid valve repair, *Staphylococcus aureus* sepsis and septic emboli; (d) 61-year-old female patient with lung hemorrhage as a result of systemic sclerosis vasculitis.

Occasionally, acute dyspnea may be caused by rare pathologies, such as permeability (non-cardiogenic) edema (Figure 7A), hypersensitivity pneumonitis (Figure 7B), septic emboli (Figure 7C), or vasculitis (Figure 7D).^37^ Permeability edema manifests on CT as multifocal extensive ground-glass opacities, sometimes confluent with a dependent distribution and atelectasis; acute hypersensitivity pneumonitis^38^ most frequently appears as a result of organic antigen inhalation as multifocal ground-glass opacities combined with ill-defined centrilobular nodules predominantly in the mid and lower lung zones and lobular areas of air trapping. Septic emboli result in multiple, peripheral nodules in varying stages of evolution, with some solid, and others cavitary or cystic, and associated peripheral wedge-shaped consolidations secondary to infarction and pleural effusion. Vasculitis may manifest as randomly distributed nodules, sometimes cavitary, multifocal consolidations and ground-glass opacities that result from pulmonary hemorrhage.^37^

**Figure 8.**
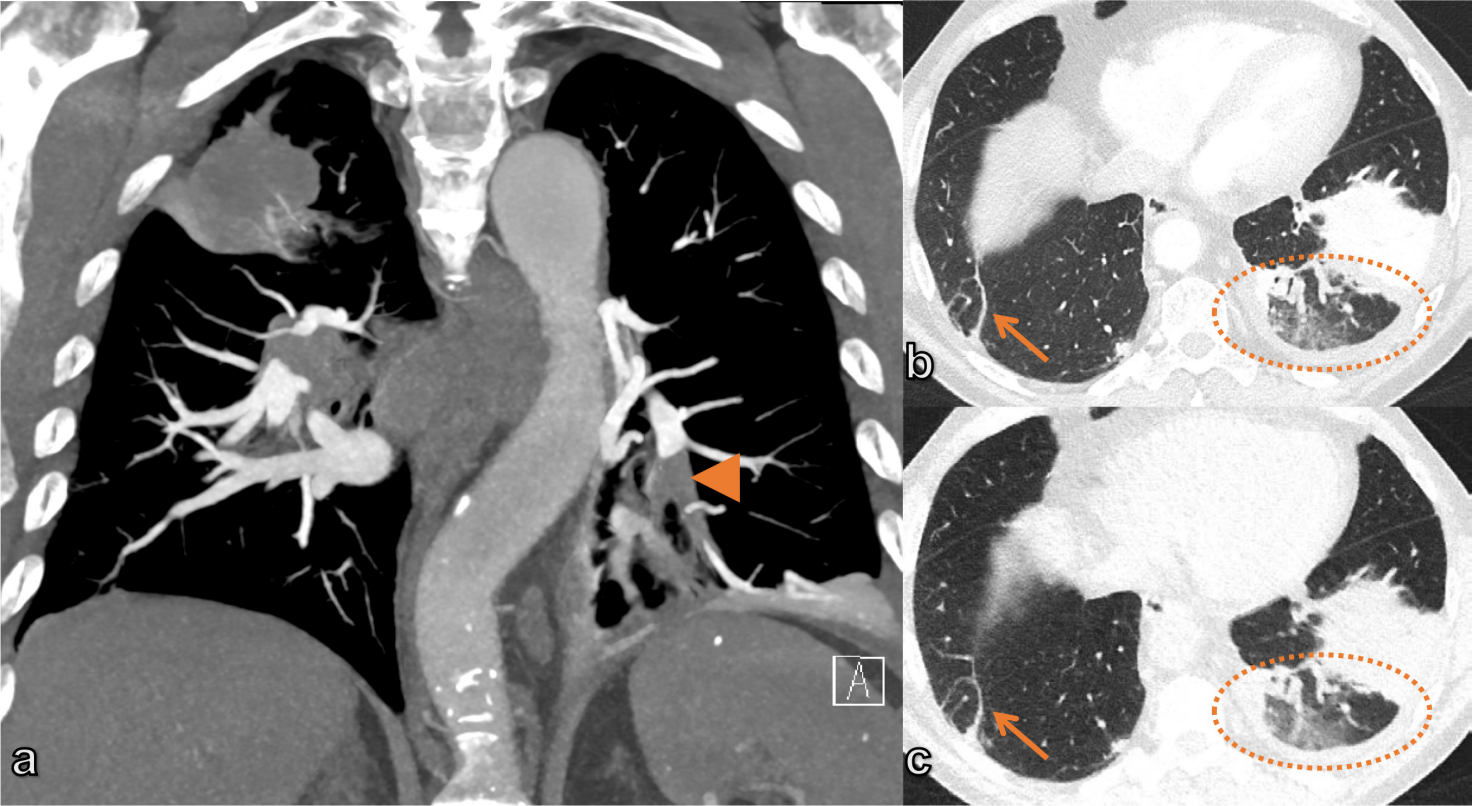
63-year-old male dyspneic patient who was referred for CTPA with suspicion of PE. The CT revealed, in addition to the segmental PE in the left lower lobe (arrowhead), a mass in the right upper lobe with pathological mediastinal lymphadenopathy (a). The patient also underwent an ultra-low-dose chest CT, which showed a good correlation with the standard CT in depicting an infarcted area of lung parenchyma in the left lower lobe (circle) and an atelectasis in the right lower lobe (arrow) (b) standard CT and (c) ultra-low-dose CT. CTPA, CT pulmonary angiography; PE, pulmonary embolism.

Pulmonary embolism (PE) is a potentially fatal disease seen in patients with dyspnea, with approximately 2% of individuals with venous thromboembolism dying due to PE.^39^ Dyspnea is the leading symptom in patients prior to cardiac arrest caused by pulmonary embolism.^40^ On CXR, pulmonary embolism can manifest with the following signs: Fleischner sign (enlarged pulmonary artery); Hampton hump (peripheral wedge-shaped opacity with the apex pointing to the hilus representing lung infarction); Westermark sign (regional oligemia distally to the site of the embolism); knuckle sign (abrupt tapering/cutoff of a pulmonary artery); right heart and azygos vein enlargement and pleural effusion. However, chest radiographs are not reliable in diagnosing PE (Table 1) and advanced imaging is commonly used for its diagnosis, with the modality of choice being the CT pulmonary angiography (CTPA).^41^ CTPA showed a pooled sensitivity of 94% and a pooled specificity of 98%^42^ and can be used as a standalone imaging test to exclude PE.^41^ It allows adequate visualization of the pulmonary arteries down to the subsegmental level. CTPA shows filling defects within the pulmonary vasculature corresponding to emboli, which may be occlusive or non-occlusive, with an acute angle to the vessel, differentiating acute from chronic emboli (Figure 6). CTPA can also assess the right ventricular size and function by the detection of right ventricle (RV) enlargement as an indicator of RV dysfunction and prognostic factor.^41^ A meta-analysis of 49 studies confirmed that, in patients with PE, an increased ratio between the right and left ventricle of more than one on CT was associated with a 2.5-fold increased risk for all-cause mortality and a 5-fold risk for PE-related mortality.^43^ CTPA can also assess right heart strain that presents as contrast reflux to the inferior vena cava, dilatation of the hepatic veins in the presence or absence of contrast agent reflux, and dilatation of the azygous system. CTPA may provide an additional or alternative diagnosis if PE is excluded (Figure 8).

Since its introduction, CTPA has been widely employed and rates of use have increased dramatically, resulting in concerns about overuse. The number of CTPA scans increased by 450% from 2004 to 2016 in the USA^44^ and the yield rate for PE from CTPA scans in the meta-analyses ranged from 3%^45^ to 13%^46^ in North America and 29% in Europe.^46^ To limit unnecessary diagnostic testing, there have been growing efforts to create and implement decision rules for PE testing that rely on risk stratification algorithms, such as the revised Geneva score or the Wells score, combined with D-dimer testing. These risk stratification-based strategies have been extensively validated and implemented into clinical practice to identify low-risk patients for whom CTPA can be safely avoided.

**Figure 9.**
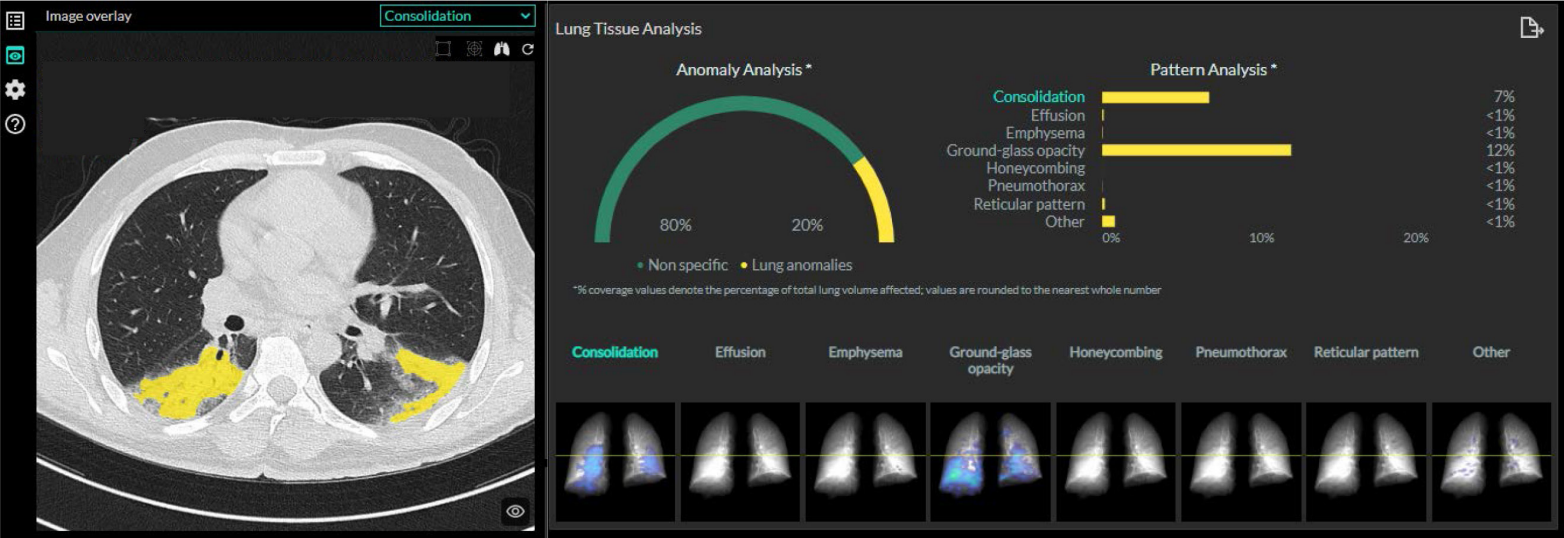
Example of a quantitative pattern analysis report regarding the percentage of lung anomalies (consolidation and ground-glass opacities) detected on a chest CT scan of a 42-year-old-patient with COVID-19 pneumonia.

One of the weaknesses of CT is radiation exposure. Efforts have been constantly undertaken to reduce the radiation to a minimum while preserving the diagnostic accuracy. Recent developments have allowed the use of ultra-low-dose CT at an effective dose of 0.05 mSv for the detection of chest pathologies in emergency settings, showing an equal or improved sensitivity compared to CXR in detecting acute chest conditions^47^ (Figure 8C). Another hot topic in medical imaging currently is the development of artificial intelligence (AI) algorithms in thoracic imaging, with the potential to improve the diagnostic accuracy and management of patients through automated detection, quantification, and classification of lung abnormalities. During the COVID-19 pandemic, a multitude of publications emerged regarding AI approaches using chest CT and CXR for the diagnosis and quantification of COVID-19 pneumonia (Figure 9), mortality risk, risk for admission to intensive care unit, disease management, or monitoring of cases.^48^

## Ultrasound

Point-of-care ultrasonography (POCUS)^49^ has become a widely used bedside diagnostic tool for patients with acute dyspnea in the ED to enable immediate therapeutic action. POCUS does not require ionizing radiation exposure and does not require the transfer of patients to radiology suites. It encompasses lung and pleura ultrasound, echocardiography, and lower extremity compression ultrasound of the deep veins.^50^ Several studies and systematic reviews have assessed the use of POCUS for the evaluation of acute dyspnea.^51,52^ Zanobetti et al demonstrated that no statistically significant differences could be found in the accuracy of POCUS and the standard ED workup for the diagnosis of acute coronary syndrome, pneumonia, pleural effusion, pericardial effusion, pneumothorax, and dyspnea from other causes. Furthermore, POCUS was shown to be more sensitive for the diagnosis of heart failure, while the standard ED workup performed better in the diagnosis of COPD/asthma and pulmonary embolism.^52^ The disadvantage of ultrasound is that it is operator dependent and requires expertise.

## Suspected cardiac origin

**Figure 10.**
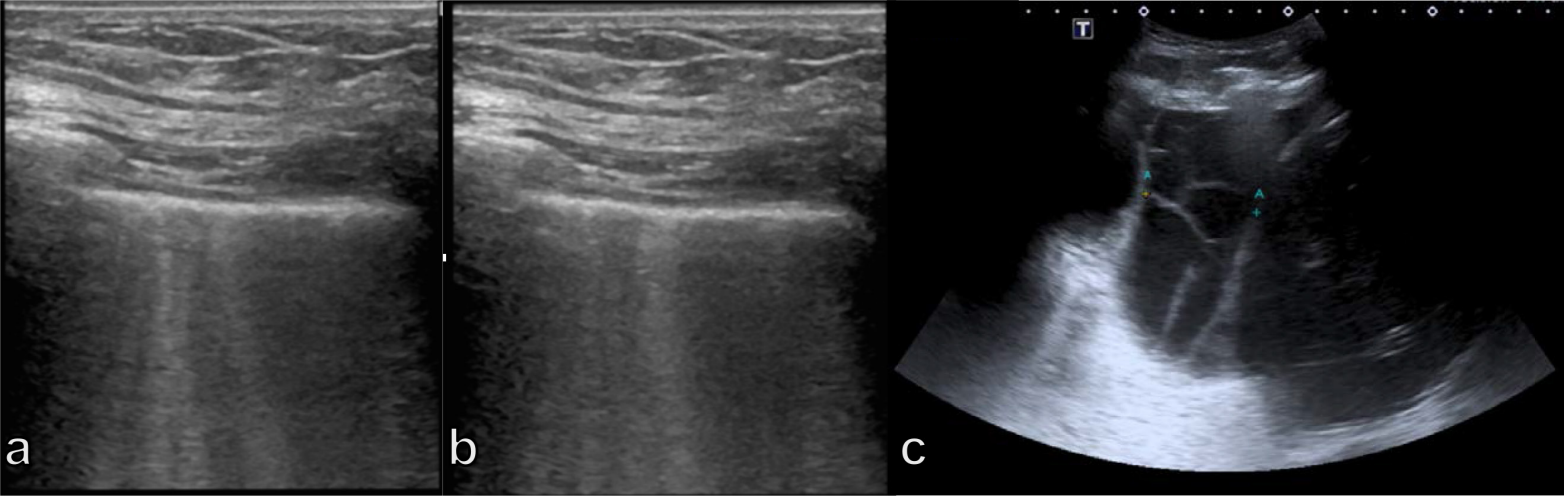
(a, b) Lung ultrasound of a 61-year-old male dyspneic patient depicting B-lines indicative of cardiogenic pulmonary edema; (c) 17-old-year male patient with complex parapneumonic pleural effusion showing septa.

In a metanalysis, lung ultrasonography (LUS) was found to be more sensitive than CXR for the detection of cardiogenic pulmonary edema in AHF and had comparable specificity.^53^ A-lines are horizontal repetition artifacts in form of echoic lines that arise from the pleura line at regular intervals indicating a normal lung surface and subpleural air, which completely reflects the ultrasound beam.^54^ Sonographic B-lines or the “comet-tail artifacts” are hyperechoic reverberation artifacts that fan out vertically from the pleural surface to the bottom of the screen without fading and move synchronously with the lung sliding (Figure 10A and B).^55^ The B-lines are generated by an air-fluid mixture, which occurs when subpleural interlobular septa surrounded by subpleural air-filled alveoli become edematous.^54^ The number of B-lines has been shown to offer a semi-quantitative measure of extravascular lung water content^56^ and to be associated with an increased risk for adverse events.^57^ Therefore, the lung ultrasound was included as a diagnostic test with which to confirm AHF in the latest European Guidelines for the diagnosis and treatment of acute and chronic heart failure.^8^

## Suspected pulmonary origin

Ultrasound has proven to be an excellent tool for the diagnosis of pleural effusion^58^ and pneumothorax,^59,60^ with superior diagnostic accuracy compared to CXR. Ultrasound diagnosis of pleural effusions is based on the visualization of fluid collections within the pleural space. Free-flowing pleural effusions accumulate in the most dependent portions of the thorax, most commonly the posterolateral costophrenic recesses and are best evaluated starting at the level of the diaphragm.^61^ Pleural ultrasound can detect physiologic amounts of fluid (5 ml), but a minimal volume of 20 ml is more reliably detected^61^ and can differentiate between simple (anechoic, usually transudative) and complex effusion (heterogeneously echogenic, with septations (Figure 10C), more often exudative).^61^ The ultrasound diagnosis of a pneumothorax relies on several indirect signs, such as the lack of lung sliding (*i.e.* the sliding of the pleural interface during respiration), the so-called lung pulse (*i.e.* pulse-synchronous movements of the pleural interface), and the visualization of the so-called lung point (*i.e.* the intermittent contact zone of the visceral and parietal pleura).^62,63^ The accuracy of ultrasound for the detection of pleural abnormalities is high, and published values include a sensitivity of 92–100% and a specificity of 93–100% for pleural effusions,^61^ and a sensitivity of 95% and a specificity of 91% for the detection of pneumothorax.^63^

**Figure 11.**
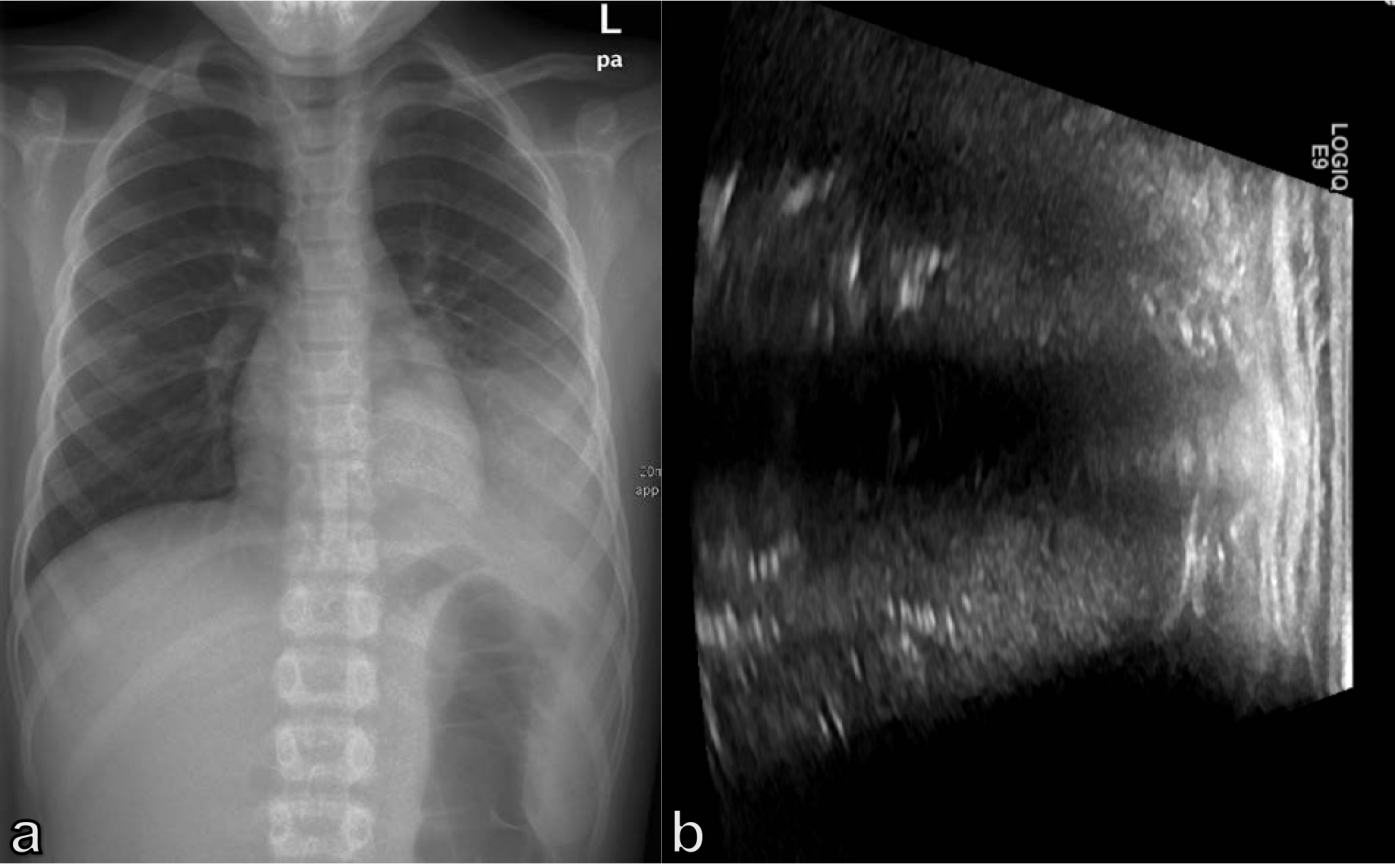
(a) CXR of a 9-year-old boy with dyspnea and pneumonia and (b) corresponding ultrasound image of the pneumonia, depicting the consolidation of the lung parenchyma with punctiform hyperechogenic spots that correspond to the air bronchograms. CXR, chest X-ray.

For the assessment of pneumonia, the majority of studies considered LUS examination to be positive if sonographic consolidations or multiple B-lines were observed.^64^ The sonographic appearance of consolidations in pneumonia includes hyperechogenic spots and tree-like structures, which indicate air in the small bronchi (Figure 11). The tree-like structures seem to correspond to air bronchograms in chest radiography.^64^ There have been extensive retrospective studies on the application of LUS in the assessment of COVID-19 pneumonia, which showed a sensitivity ranging from 68 to 97% and a specificity ranging from 21 to 79% for LUS.^65^ LUS is also an ideal imaging method for the pediatric population, because it does not expose patients to radiation. It was shown to have significantly better sensitivity, with a specificity similar to that of chest radiographs for the diagnosis of pediatric CAP,^66^ supporting LUS as an imaging alternative in childhood pneumonia.^67^

## Conclusion

Identifying the cause of dyspnea often requires a targeted application of imaging modalities (Table 2) to establish the correct diagnosis. The appropriate choice of imaging demands an in-depth knowledge of the indications, findings, and limitations of each particular imaging method.

**Table 2.** The appropriate choice of imaging modalities in conditions that can accompany acute dyspnea of suspected cardiac and pulmonary origin

Condition	Imaging modality
**Dyspnea of suspected cardiac origin**	
Acute coronary syndrome (low- and intermediate-risk)	**CCTA**
Cardiogenic pulmonary edema	**CXR / TTE / LUS**
Pericardial effusion	**TTE / CXR / MRI** (in case of constrictive pericarditis)
Pericardial tamponade	**TTE / CTA of the aorta**
**Dyspnea of suspected pulmonary origin**	
Pneumothorax	**LUS / CXR**
Tension pneumothorax	**Clinical signs / LUS / CXR**
Pleural effusion	**LUS / CXR**
Pneumonia – immunocompetent patients	**CXR / CT / LUS**
Pneumonia – immunocompromised patients	**CXR / CT** (if high clinical suspicion, but CXR normal or equivocal)
Pulmonary embolism	**CTPA**
Asthma exacerbation	**CXR**
COPD exacerbation	**CXR / CT**

CCTA, coronary CT angiography; COPD, chronic obstructive pulmonary disease;CT, computer tomography; CT, computer tomography;CTA, CT angiography; CTPA, CT pulmonary angiography; CXR, chest radiograph;LUS, lung ultrasound; TTE, transthoracic echocardiography.
